# Strategies to Increase Vaccinations in Adult Cancer Patients: A Systematic Review

**DOI:** 10.3390/vaccines13090964

**Published:** 2025-09-11

**Authors:** Giuseppina Lo Moro, Federica Golzio, Sara Claudia Calabrese, Giacomo Scaioli, Alessandro Basile, Roberta Siliquini, Fabrizio Bert

**Affiliations:** 1 Department of Public Health and Pediatric Sciences, University of Turin, 10126 Turin, Italy; federica.golzio@unito.it (F.G.); saraclaudia.calabrese@unito.it (S.C.C.); giacomo.scaioli@unito.it (G.S.); alessandro.basile@unito.it (A.B.); roberta.siliquini@unito.it (R.S.); fabrizio.bert@unito.it (F.B.); 2 Health Directorate, Azienda Ospedaliera Universitaria City of Health and Science of Turin, 10126 Turin, Italy; 3 Collegium Medicum, University of Social Sciences, 90-113 Lodz, Poland

**Keywords:** cancer patients, vaccination, systematic review

## Abstract

Background/Objectives: Although vaccinations are a priority for patients with cancer, achieving high coverage remains challenging. Evidence on effective strategies in oncology settings is still limited. This systematic review aimed to identify interventions to improve vaccination uptake or reduce hesitancy among cancer patients. Methods: A systematic search was conducted in PubMed, Embase, and Scopus, including studies published up to the end of 2023. The protocol was registered in PROSPERO (CRD42024511008). Results: Out of 10,927 non-duplicate records, 15 studies describing unique interventions were included. All studies were published between 2011 and 2022, primarily conducted in Europe/UK (40%) and in North America (40%). The most common study design was pre-post (60%), and 33.3% included a control group. Most interventions were multi-component (60%) and were classified into three main categories: educational materials/campaigns (46.7%), reminders (40%), and patient counselling (33.3%). Additional components included guideline development in two studies. Some studies also highlighted the importance of specific key figures, such as dedicated professionals, general practitioners, and pharmacists. Interventions mainly targeted patients (40%), with 33.3% addressing both healthcare professionals and patients and 26.7% professionals only. They most frequently concerned vaccinations against influenza and pneumococcal disease (26.7%), pneumococcal disease alone (26.7%), or Coronavirus Disease 2019 (COVID-19) (26.7%). Vaccination uptake was the primary outcome in 86.7% of studies, with 66.7% reporting significant improvements. Conclusions: This review identified a variety of strategies, with education, reminders, and counselling as key components. Multicomponent interventions and those involving both patients and providers were most promising. However, methodological limitations and limited generalizability highlighted the need for more rigorous research.

## 1. Introduction

According to the Global Cancer Observatory, the incidence of new cancer cases in 2022 was almost 20 million worldwide [1]. Patients with cancer have a weakened immune system due to both the disease itself and its specific therapies. Consequently, these patients have an increased risk of infections and related complications [2,3]. In particular, a recent U.S. study showed that the risk of death from infections among cancer patients was three times higher than in the general population [4]. Infections are the leading cause of morbidity and mortality among cancer patients, resulting in frequent and prolonged hospitalizations [5,6]

Vaccination is an effective strategy to reduce the risk of infection-related complications and deaths [7]. Evidence consistently showed that immunization can enhance survival. For instance, in the ASCO COVID-19 registry, patients with cancer who had not received a SARS-CoV-2 vaccine had a 1.60-fold higher risk of death (95% CI 1.26–2.04) compared with vaccinated individuals [8]. A multicenter study reported that influenza vaccination improved overall survival (OS) among patients receiving immune checkpoint inhibitors, with a hazard ratio (HR) of 0.75 (95% CI 0.62–0.92) and increased disease control rate (OR 1.47, 95% CI 1.11–1.96) [9]. Pneumococcal vaccination also significantly improved OS: in the study by Li et al. the 7-year OS rates were 47.5% in the vaccinated group versus 42.3% in the unvaccinated group (log-rank *p* = 0.0003) [10]. In addition to clinical benefits, vaccinations have proven cost-effective in immunocompromised populations. For example, recombinant zoster vaccine in 19,671 U.S. hematopoietic stem cell transplant recipients resulted in total societal cost savings of USD 0.1 million and 109 incremental quality-adjusted life years (QALYs) compared with no vaccine [11]. Adding PCV13 to PPSV23 in adults with immunocompromising conditions yielded 1,360 discounted QALYs and net savings of USD 5.2 million [12]. Finally, influenza vaccination in working-age cancer patients was also highly cost-effective, with an incremental cost-effectiveness ratio of only USD 224 per QALY gained [13].

Indeed, although the immune response of oncological patients can be persistently suppressed, they can still reach seroprotection in many cases thanks to vaccines, thus making some of the most common infections in cancer patients vaccine-preventable [14]. In 2024, the American Society of Clinical Oncology (ASCO) published vaccination guidelines for adult patients with cancer, recommending a broader range of vaccines for this population and highlighting that vaccine strategies may differ from those for the general population [15].

Although vaccinations are a priority for patients with cancer, available data highlighted the difficulty in achieving high coverage rates in this population. For example, despite a long-standing target of at least 75% influenza vaccination coverage among at-risk groups including cancer patients [16], evidence from various countries consistently showed substantially lower uptake in this population [17,18]. In this context, it is essential to recognize that patients should not be the sole actors in the vaccination decision. Patients with immunocompromising conditions seem to be particularly concerned about vaccine-related side-effects, trust in the effectiveness, and fear of deterioration of their underlying disease [19]. As a result, healthcare professionals (HCPs) play an important role in the patients’ vaccination decision-making and collaboration between specialists and general practitioners is crucial [20]. Indeed, vaccination uptake is strongly influenced by HCP recommendations [19,21]. A recent review on the general population highlighted the value of behavioral strategies, such as tailored education, reminders, and direct communication, in addition to provider recommendations [22]. However, evidence on effective strategies in oncology settings remains limited and, in line with ASCO guidelines, vaccination should become a central component of cancer care [15].

Therefore, the present systematic review aimed to identify and synthesize existing interventions to improve vaccine uptake or reduce hesitancy among cancer patients and cancer survivors. In addition, this review sought to describe the efficacy of these strategies, providing an overview of the current evidence in this population.

## 2. Materials and Methods

This review was conducted according to the latest version of the Preferred Reporting Items for Systematic reviews and Meta-Analyses (PRISMA) guidelines [23]. The protocol of this review was registered in the International Prospective Register of Systematic Reviews (PROSPERO) (CRD42024511008). Preliminary findings from this review were presented as a poster at the 57th National Congress of the Italian Society of Hygiene, Preventive Medicine and Public Health (SItI) [24].

### 2.1. Eligibility Criteria

Methods and search strategy were planned in advance, after a preliminary literature analysis. The main review question was “Are there strategies to reduce vaccine hesitancy and/or improve vaccine adherence among cancer patients and/or cancer survivors? Are these strategies effective?” and the strategy was developed according to the PICO framework. About “Population”, all the studies about adult oncological patients were considered. We defined “adult” as any patient aged 18 years or older and “oncological patient” as any patient currently under treatment (chemotherapy, radiotherapy, and every other cancer therapy), newly diagnosed patients, patients currently in follow-up, and cancer survivors who had completed follow-up. Regarding “Intervention”, all the strategies or interventions that aimed to increase vaccine uptake or reduce vaccine hesitancy among cancer patients/survivors or enhance vaccine recommendations by HCPs were considered. Thus, the target of the intervention could be both patients and/or HCPs, as long as the ultimate broad aim of the intervention is to increase vaccine coverage among the oncological population. Considering “Comparison”, studies with any comparison were considered, including those without a control group. Lastly, for “Outcome”, studies with any primary outcome evaluating the efficacy of strategies to reduce the vaccine hesitancy and/or to improve vaccination adherence were considered; for instance: vaccine uptake/coverage in the oncological population, vaccine recommendations by HCPs, patients’ willingness to get vaccinated, knowledge on vaccinations among oncological patients/survivors and/or HCPs.

We considered eligible only peer-reviewed research articles written in English, as this is the only language fully understood by all Authors in addition to their native language. We did not consider any restriction about country or publication year. In addition to the criteria defined by the PICO framework, we included any primary study with original data evaluating an intervention, regardless of design. Eligible study types included randomized controlled trials (RCTs), quasi-experimental studies (with or without control groups), case–control studies, and cohort studies. Research protocols, letters to the editor, and commentaries were excluded because they do not provide original data. According to this criterion, systematic and narrative reviews were excluded from data extraction. However, pertinent reviews were retained during the study selection to identify additional eligible primary studies in the bibliography.

### 2.2. Information Sources and Search Strategy

We performed the primary search in three different databases (PubMed, Embase, and Scopus). PubMed and Embase were selected as they are considered essential databases for biomedical systematic reviews [25], while Scopus was added as a multidisciplinary source offering broad coverage and frequent use in evidence synthesis [26,27]. A predetermined search query was conducted on 24 November 2023, with adjustments applied to each database. Then, we set alerts to collect studies published by 31 December 2023. During a subsequent step, we noticed that the search query did not include some results of interest, so we decided to launch a second query with new keywords on 20 February 2024. The results of both queries and alerts were uploaded to the web application Rayyan of the Qatar Computing Research Institute [28].

The search strategy combined free-text terms and controlled vocabulary terms for three main concepts: vaccination (e.g., vaccine*, immuniz*, immunis*), cancer (e.g., cancer*, malignan*, tumor*, tumour*, neoplasm*), and vaccine hesitancy or uptake (e.g., hesit*, uptake*, coverage*, refus*). All the queries we used are shown in the Supplementary File (Supplementary File S1).

### 2.3. Selection Process

First, four Authors (G.L.M., A.B., S.C.C., F.G.) manually removed duplicates using Rayyan. The Authors proceeded to screen the title and abstract of all retrieved records. As a training, the Authors initially reviewed 5% of the papers. After a consensus-based discussion, the team proceeded to screen all the remaining titles and abstracts. Then, the Authors screened the full texts of the papers selected in the previous steps. Only after full-text selection, the reference lists of included studies were screened to identify additional relevant publications. The Authors also screened the reference lists of systematic reviews related to vaccines, cancer patients, and vaccination strategies, provided these reviews had been deemed relevant after full-text screening.

Every step of study selection was performed by at least two Authors independently and in duplicate and followed by a consensus-based discussion with the entire team to solve conflicts, except for the duplicate deletion.

### 2.4. Data Collection Process and Data Items

Data extraction was performed using Microsoft Excel. The extracted data were: first author, year of publication, language, country; characteristics of the study, e.g., study design, sample size, presence of a control group, setting, length of the study and year of the study; characteristics of participants; characteristics of the intervention (and of the control group, if applicable), e.g., vaccine used, component of the intervention; outcome measures and results; presence of any declared conflicts of interest and funding sources; any other valuable comment. Following a discussion among all members of the research team, interventions were classified into categories and their main components were identified, to provide a narrative synthesis of the findings.

Every step of data extraction was performed by at least two Authors blinded to each other and followed by a consensus-based discussion with the entire team to solve conflicts.

### 2.5. Study Risk of Bias Assessment

To assess the risk of bias of the RCTs, we used the Cochrane risk-of-bias tool for randomized trials (RoB 2) version 2 [29]. For the risk of bias in non-randomized studies, we used the revised Joanna Briggs Institute (JBI) critical appraisal tool [30]. At least two Authors independently rated each paper, and disagreements were solved by a consensus-based discussion with the entire team.

### 2.6. Synthesis Methods

We conducted a narrative synthesis of the interventions. The synthesis focused on the types of strategies used and their reported primary outcomes and primary results. Due to heterogeneity in study designs, target populations, intervention components, and outcome measures no quantitative synthesis was performed. Along with a narrative synthesis, Summary of Findings tables were created to present the key characteristics and results of each included study.

### 2.7. Effect Measures, Reporting Bias Assessment and Certainty Assessment

Due to the heterogeneity of interventions and as no quantitative synthesis was conducted, effect measures were not pooled across studies. Accordingly, no formal assessment of reporting bias was performed. We assessed the certainty of the evidence using the Grading of Recommendations Assessment, Development, and Evaluation (GRADE) approach [31]. The evaluation was performed by one reviewer and independently verified by a second reviewer. Given the heterogeneity of study designs, interventions, and outcomes, we restricted the assessment to the most frequently reported and relevant outcomes, as agreed upon by the review team after discussion. These included pneumococcal vaccination coverage (n = 10 studies), influenza vaccination coverage (n = 4), and COVID-19 vaccination coverage (n = 2).

## 3. Results

### 3.1. Study Selection

**Figure 1:**
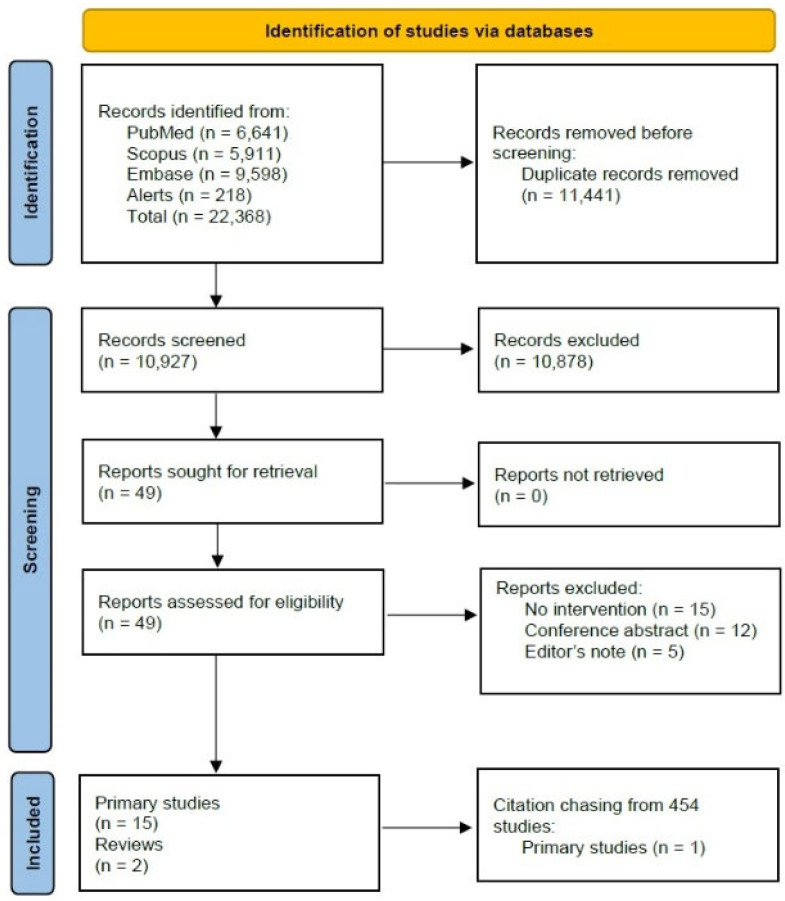
PRISMA Flow diagram.

We screened 10,927 not-duplicate records. Figure 1 summarizes the study selection flow. The complete flow diagram, including the two search queries separately, is available in the Supplementary Material (Supplementary Figure S1). Once the full-text selection process was completed, 18 articles were considered eligible; two of these papers were reviews [32,33] (used for the backward citation chasing). Of the 16 remaining papers, two articles were by the same Author and described the same intervention and results [34,35]. One of these papers [35] was a research letter and, in our results, we only considered the more comprehensive study [34]. Thus, a total of 15 articles and unique interventions were included.

More than half of the studies (53.3%) declared no funding and only 2 studies (13.3%) did not report any information. Considering conflict of interests, 86.7% disclosed no conflicts and only 1 study (6.7%) did not have information about it. (Supplementary Table S1)

### 3.2. Study Characteristics

All included studies were conducted between 2011 and 2022 (published between 2015 and 2023); 47% of studies were active in 2021. The studies occurred primarily in European/UK (40%) [34,36,37,38,39,40] and North American (40%) [41,42,43,44,45,46] settings; remaining studies were conducted in India, Israel, and Turkey [47,48,49]. The most frequently used study design was pre-post (60%) [34,36,37,38,40,41,43,44,48], while only two studies were RCTs [46,49]. (Table 1) Some form of control group (usual care) was present in 33.3% of the studies [34,39,45,46,49] (Table S1).

The studies varied in length from the shortest (17 days [41]) to the longest (28 months [38]), with 60% of studies lasting at least 12 months [34,38,42,44,45,46,47,48,49]. Most interventions were directly targeted to patients (40%) [37,39,40,46,47,49], 33.3% were targeted to both HCPs and patients [41,42,43,45,48], and 26.7% only HCPs [34,36,38,44]. The number of participants in the interventions ranged from 10 HCPs [42] to 3603 patients [47]; however, 26.7% of the studies did not report participant numbers. More than half of the studies (60%) did not consider only a specific type of cancer. Three studies (18.8%) focused on onco-hematologic diseases [39,43,47], 2 studies (12.5%) on gynecologic cancers [42,46], 2 studies on gastroenterological cancers (12.5%) [37,46], and 1 on lung cancer (6.3%) [46]. Only 1 study [45] also considered pediatric patients. Most interventions concerned vaccinations against flu and pneumococcal disease (26.7%) [36,38,42,46], vaccinations against pneumococcal disease (26.7%) [43,44,48,49] or Coronavirus Disease 2019 (COVID-19) (26.7%) [39,40,41,47]. Only one study (6.7%) targeted herpes zoster and hepatitis b in addition to flu and pneumococcal disease [34]. In one study (6.7%) [37], although the evaluation was focused on flu and pneumococcal vaccinations, the intervention also addressed diphtheria-tetanus-poliomyelitis, hepatitis b, and papillomavirus (HPV) vaccinations.

A large proportion of the interventions had multiple components (60%) [36,38,39,42,43,44,45,48,49]. Only 2 interventions (13.3%) were delivered entirely online [40,41]. The interventions were grouped into three main categories: educational materials and campaigns (included in 46.7% of interventions [36,40,41,42,44,45,49]), reminders (in 40% [34,38,39,43,45,48]), and patient counselling (in 33.3% [37,39,46,47,49]). Among additional components, the development of specific vaccination guidelines or protocols was a recurring element, implemented in two interventions [36,38]. Most studies (86.7%, n = 13) assessed patient vaccination rates as the primary outcome [34,36,37,38,39,42,43,44,45,46,47,48,49], even when the intervention was primarily directed at HCPs. Among these, 66.7% (n = 10) reported statistically significant improvements in vaccination rates. Only three studies (20%) [40,41,49] evaluated other types of outcomes, such as vaccine intention, and changes in beliefs, attitudes, or knowledge. Information about the components is reported in Table 2, while the information about primary outcomes and results is presented in Table 3.

In conclusion, the included studies were highly heterogeneous, precluding quantitative synthesis. This heterogeneity was largely driven by substantial differences in intervention composition and target populations. For instance, focusing on the three most frequently reported outcomes, only in two cases did the studies with pneumococcal vaccination coverage as the primary outcome (n = 10) involve interventions that could be classified into similar categories; the remaining cases differed in their components. For influenza (n = 4) and COVID-19 vaccination coverage (n = 2), interventions were entirely distinct in composition. Differences in delivery modes, target groups, and outcome measures further limited comparability and made pooling of results infeasible.

### 3.3. Results Synthesis

#### 3.3.1. Educational Materials and Campaigns

Educational materials and campaigns were the most frequent component, being present in 7 interventions (46.7%) [36,40,41,42,44,45,49]. Education was targeted at patients (n = 3) [40,45,49], HCPs (n = 2) [36,44], or both HCPs and patients (n = 2) [41,42]. The majority of interventions included other components in addition to education (n = 5) [36,42,44,45,49].

In 5 interventions, it was the sole component [40,41] or the main component [36,42,44]. All these studies had a quasi-experimental design. The two studies that included exclusively an educational component were both focused on COVID-19 vaccination and delivered online in different formats. One was an educational webinar targeting both patients and HCPs aimed at improving knowledge about COVID-19 vaccination and reducing hesitancy [41], while the other was an interactive web tool offering individualized information on vaccine benefits and risks for patients [40]. Although both interventions reported improvements in vaccine intention, neither study conducted statistical analyses to confirm significance. However, Kelkar et al. [41] did report a statistically significant change in beliefs and perspectives on COVID-19 vaccines.

Among the multi-component interventions in which education was the main component [36,42,44], results were mixed. Overall, the training aimed to update HCPs on vaccinations and encourage them to actively recommend vaccines to patients. When HCP education was combined with the recruitment of an advanced practice nurse practitioner (APRN) to act as a safety net, reviewing patients’ vaccination status and prescribing vaccines when oncologists had not, a significant increase in patient vaccination coverage was observed [44]. Notably, the APRN’s involvement was not part of the original design but was introduced after an interim review, as oncologists were not addressing vaccination despite training [44]. When HCP training sessions were accompanied by the development of a vaccination protocol validated by infectious disease specialists [36], no significant changes in coverage were observed. The combination of HCP education, patient education (i.e., information brochures), pre-printed prescriptions to improve the ease of prescribing vaccines for physicians, and organizational changes such as an in-house vaccination program [42] led to large increases in coverage although no inferential analyses were conducted by the Authors.

#### 3.3.2. Reminders

Reminders of any kind (e.g., text messages, telephone calls, personalized letters, pop-up alerts) were a component in 6 interventions (40%) [34,38,39,43,45,48]. Reminders were targeted at patients (n = 1) [39], HCPs (n = 2) [34,38], or both HCPs and patients (n = 3) [43,45,48]. Most interventions included other components beyond reminders (n = 5) [38,39,43,45,48].

In 5 interventions, reminders were the only component [34] or the main component [39,43,45,48]. Most studies had a quasi-experimental design, while one was a natural experiment [45]. Considering patient reminders, most interventions (n = 3) included letters, text messages, phone calls, or emails [39,43,48], while one involved posters [45]. As for HCP reminders, an alert in the Electronic Medical Record (EMR) was the most frequent action (n = 3) [43,45,48], while also physical cards or charts were used in two cases [34,45]. All these interventions produced statistically significant improvements in vaccination rates.

The single-component intervention [34] could be classified as both an HCP reminder and a form of protocol provision. It consisted of a card listing vaccination recommendations for cancer patients, specifically designed to remind and guide general practitioners (GPs). Patients acted as intermediaries, receiving the card from their oncologist or hematologist and delivering it to their GP. Vaccination coverage significantly increased more in the intervention group (+21.5pp) than in the control group (−5.8pp).

Three interventions included reminders both for patients and for HCPs [43,45,48]. In the intervention by Church et al. [43], providers from a virtual clinic entered vaccine orders in the EMR as reminders for HCPs, documented the rationale, and involved the primary care team. Moreover, they sent letters as reminders to patients. To isolate the effect of the virtual clinic from a prior national clinical reminder, time-to-vaccination curves were compared, showing a significant difference in favor of the virtual clinic. In the work by Grivas et al. [45], multiple HCP reminders were implemented, including “screen pop-ups” in the EMR, laminated reminders placed in the paper chart, and visual cues (e.g., stickers) to encourage discussion and vaccine ordering. Reminders for patients were posted in clinic areas near elevators, alongside educational materials shared via the institution’s website and social media. The intervention led to a significant increase in vaccination. Shapiro et al. [48] described an intervention during which HCPs received EMR alerts, prompting vaccine prescriptions, and patients were actively reminded through their digital health record, text messages, and emails every six months. Additionally, preapproval requirements for PCV13 were waived. Vaccination rates significantly increased for both PCV13 and PPSV23.

Lastly, in the intervention by Narinx et al. [39], patients received repeated personalized reminders via letters, phone calls, and messages. In addition, the intervention also included counselling during hospital visits and direct access to healthcare professionals for questions. The vaccination coverage in the intervention group was significantly higher than in the general population.

It is also worth noting the sixth intervention that included reminders, although not as a primary component [38]. This was a multi-component intervention targeting HCPs, with its core element being the development of vaccination guidelines. These were disseminated to hospital practitioners via email and internal presentations, and to local primary care physicians via email. Additional components included summary posters for HCPs placed in clinic rooms and a letter for patients to deliver to their primary care providers. Pneumococcal vaccination rates increased significantly following the intervention, whereas influenza vaccination rates (although consistently around 70%) did not change significantly.

#### 3.3.3. Patient Counselling

Patient counselling was included in 5 interventions (33.3%) [37,39,46,47,49]. Only two interventions [39,49] included additional components. Vaccination counselling was performed by physicians (n = 2) (infectious diseases specialist [37], oncologist [47]), pharmacists (n = 2) [46,49], and unspecified HCPs during hospital visits (n = 1) [39].

In 4 interventions, patient counselling was the only component [37,46,47] or the main component [49]. Two studies had a quasi-experimental design [37,47]: one of them showed a significant improvement in vaccination rates [37], while the other described a post-intervention analysis without inferential statistics [47]. Two were RCTs [46,49] and both reported a significant improvement in vaccination rates. Considering the single-component interventions, Sitte et al. [37] implemented consultation with a senior infectious diseases specialist. During the visit, the timing of live attenuated vaccines and the updating of various vaccinations (including diphtheria–tetanus–poliomyelitis, HBV, influenza, and pneumococcal vaccines) were discussed, leading to a significant increase in pneumococcal vaccination rates. In the study by Ganju et al. [47], patients were actively counseled by treating oncologists regarding the available COVID-19 vaccines during clinic visits or hospital admissions. At least one dose of COVID-19 vaccine was administered to 86% of the patients and 67% received both doses. Nipp et al. [46] evaluated an intervention based on a pharmacist’s visit. The pharmacist met with patients and collected a detailed vaccination history, recommended guideline-indicated vaccinations, and communicated these recommendations to the oncology team. Patients in the intervention had higher rates of newly received vaccinations for pneumonia and influenza compared with those receiving usual care.

Last, Ozdemir et al. [49] evaluated a pharmacist-led intervention. The intervention group received counselling about immunization, pneumococcal disease and vaccines, and misconceptions about vaccines; they also received educational materials on these topics. The pneumococcal vaccine was recommended according to guidelines and patients in the intervention group were followed up by phone calls 3 months after the intervention. The intervention group achieved significantly higher vaccination rates and demonstrated greater knowledge and more favorable attitudes toward vaccination.

### 3.4. Risk of Bias in Studies and Certainty of Evidence

Among quasi-experimental and natural experimental studies (n = 13), two frequent sources of high risk of bias were identified: the absence of an independent control group (present in only 23.1% of studies; [34,39,45]) and the lack of repeated outcome measurements before and after the intervention (reported in only 7.6% [42]) (Table S2). As for the two RCTs [46,49], they were judged at low risk of bias for missing outcome data, measurement of the outcome, and selection of the reported result. Both RCTs were at high risk of bias for the randomization process domain. One study [46] was at low risk of bias regarding deviations from intended interventions, while the other [49] raised some concerns in this domain. Overall, both RCTs were rated as having a high risk of bias (Figure S2).

Due to the substantial variation across interventions, in most cases the certainty of the evidence using the GRADE assessment was based on a single study. Only in two instances (both concerning the primary outcome of pneumococcal vaccination coverage) two papers reporting interventions with similar components were considered together in the assessment. Across all three outcomes, the certainty of evidence was rated for each intervention as very low. This was predominantly due to risk of bias and imprecision (Table S3).

## 4. Discussion

This systematic review identified strategies to improve vaccine uptake or reduce vaccine hesitancy among cancer patients. Despite heterogeneity in settings, populations, and intervention designs, several key themes emerged. Interventions fell into three main categories: education, reminders, and counselling.

Educational materials and campaigns were the most frequently used components across interventions. These strategies targeted either HCPs, patients, or both. Among multi-component interventions where HCP education was the primary element, results were mixed. Interestingly, in one study [44], provider training combined with the subsequent addition of an APRN to review vaccination status and prescribe vaccines led to a significant increase in coverage. The APRN was introduced after it became clear that oncologists were not addressing vaccination despite prior training. This underscored a critical limitation of education-only interventions: knowledge alone may not translate into action without structural or workflow support. Indeed, when HCP education was paired solely with the development and dissemination of a vaccination protocol [36], no statistically significant improvements in vaccination rates were observed. This was the only study in the entire review where statistical testing was performed and failed to show a benefit. In contrast, when HCP education was combined with patient-directed education, it also led to positive outcomes [42]. These findings suggest that educating oncology professionals is a valuable foundation but must be integrated into broader strategies that address other barriers. Simply providing information or guidelines may not be enough to overcome competing priorities, uncertainty about clinical roles, or lack of time. These findings may also reflect that changing human health-related behavior is difficult and takes time [50]. Ensuring that someone within the care team is explicitly tasked with reviewing vaccination status and initiating vaccination, such as an APRN, may thus increase continuity of care.

Reminders represented another key component and were consistently associated with significant improvements in vaccination uptake. Consistently, in other context, reminder-based interventions have proven effective in increase vaccine adherence among at-risk populations, especially when integrated into multi-modal strategies [51,52,53]. These interventions employed a variety of formats depending on the target: patient-directed reminders most commonly involved letters, text messages, phone calls, or emails. For HCPs, reminders were often embedded into clinical workflows through EMR alerts. These results aligned with findings in other populations, such as the study of Morgan et al. [54], who found increased vaccine uptake in prenatal care, and the one of Ruffin et al. [55], who found significant increases in HPV vaccine uptake after the introduction of a reminder system. Moreover, our review highlighted how reminders can be effectively used not only within hospital systems but also to engage primary care providers and GPs, who, despite playing a key role in delivering vaccines to cancer patients, especially in outpatient and survivorship settings, are often neglected in intervention planning and discussions focused mainly on specialists. The importance of the primary care physician is also evident in other studies, such as the study by Monier et al. targeting cancer patients, in which vaccine information provided by the primary care physician was a factor significantly associated with higher vaccination rates [56].

Patient counselling was used in one-third of interventions and was consistently associated with improved vaccination uptake. In the two RCTs, counselling interventions were delivered by pharmacists, providing the most robust evidence. Our results are consistent with prior literature: the efficacy of counselling and recommendations has already been proved in other populations [57,58,59]. Counselling is especially important for cancer patients, as previous studies have shown that oncologist recommendations strongly influence vaccine acceptance [21,60]. However, our review suggested that effective counselling can also be delivered by other professionals, such as pharmacists or infectious disease specialists, broadening the scope of who can successfully support vaccine decision-making in oncology care. Integrating patient counselling into oncology workflows, especially via pharmacists, emerged as a promising model. Pharmacists are accessible, knowledgeable about medication, and capable of reinforcing provider recommendations without adding to oncologists’ workload. This approach may be particularly valuable in settings where oncologists have limited time or competing priorities. Moreover, qualitative research supported the view that pharmacists are well suited to this role, offering trusted, patient-centered dialogue that can clarify concerns and provide vaccine recommendations [61]. Lastly, according to research beyond cancer care, pharmacist’s involvement in immunization programs showed positive effects not only in increasing vaccine uptake but also in other outcomes such as patient satisfaction [62,63].

Furthermore, additional important considerations emerged. The most commonly targeted vaccines were those against influenza, pneumococcal disease, and COVID-19. However, these represent only a portion of the immunizations currently recommended for cancer patients. According to the ASCO guidelines published in 2024 [15], additional vaccines, e.g., those against herpes zoster, respiratory syncytial virus (RSV), hepatitis B, and HPV, should also be considered, depending on patient eligibility. The limited focus on a narrow set of vaccines in the included interventions likely reflected the period in which the studies were conducted, prior to the broader recommendations outlined by ASCO. This highlights an opportunity for future research to explore interventions addressing a wider range of vaccines and to assess the feasibility of multi-vaccine delivery strategies within oncology care. It is also worth noting that none of the included studies tested innovative interventions targeting HCPs, such as mobile apps, serious games, or role-play simulations, approaches that have shown promise in other HCP subgroups [64]. Exploring these strategies in oncology settings could offer new ways to engage providers. Lastly, the importance of multi-component interventions has been well supported by existing literature, [51,65,66]. These studies also emphasized that tailoring interventions to the specific characteristics and needs of the target population may further enhance their impact [51,65,66]. This supports the need for oncology-focused strategies that combine multiple actions and address the unique needs of cancer patients.

Several limitations should be considered when interpreting the results of this review. First of all, there was a wide heterogeneity among the included studies, limiting possible quantitative analyses. The overall methodological quality of the studies was low: most were quasi-experimental, with few including independent control groups or pre-post comparisons. Only two studies were randomized controlled trials, and both were rated at high risk of bias, particularly in the randomization process. Indeed, considering the overall quality of evidence, our findings highlighted a very low quality. This does not necessarily indicate lack of efficacy but rather reflects the limited robustness, heterogeneity, and methodological weaknesses of the available studies. The outcomes were mostly limited to vaccination coverage, the specific contribution of each component within multi-component interventions could not be determined, and the included studies generally did not explore whether intervention efficacy varied according to patient characteristics. Moreover, long-term effects and sustainability of the interventions remained largely unexplored. Another limitation concerned the generalizability of findings. The majority of studies were conducted in Europe and North America, with limited representation of low- and middle-income settings. Considering our Methods, we acknowledge that limiting the search to three databases and including only papers written in English may have led to the exclusion of some relevant records.

In conclusion, future studies should prioritize the development and evaluation of rigorously designed interventions, ideally through high-quality randomized controlled trials, to strengthen evidence in this field. Research should expand beyond influenza, pneumococcal disease, and COVID-19 vaccines and particular attention should be given to multi-component, oncology-tailored approaches that integrate educational, reminder-based, and counselling strategies, while exploring innovative digital solutions to engage both HCPs and patients. To effectively integrate the roles of oncologists, pharmacists, infectious disease specialists, and primary care providers, future research should also investigate resource needs, feasibility, and barriers, which are often highly context-specific. Moreover, studies should assess the differential impact of interventions across diverse patient subgroups, e.g., by cancer stage, and should evaluate long-term sustainability and cost-effectiveness. Another important direction is to explore strategies to protect cancer patients from misinformation and false rumors spread by anti-vaccination movements. Finally, extending research to low- and middle-income countries will be essential to ensure equitable access to vaccination and to generate context-specific evidence relevant to different healthcare systems.

## 5. Conclusions

This systematic review highlighted a range of strategies mainly aimed at improving vaccine uptake among cancer patients. Interventions fell into three main categories (education, reminders, and counselling) all of which showed potential, particularly when combined. Educational initiatives targeting oncology professionals were common but appeared most effective when paired with other changes, such as designating a team member to oversee vaccinations or including patient education. Reminders consistently improved coverage and were also effective when they included primary care providers, a group often overlooked despite their central role in vaccine delivery. Patient counselling also showed positive outcomes and may be successfully delivered by a range of professionals, including pharmacists. However, most interventions focused on only a few vaccines and there were issues in the generalizability of results and in the methodology of the studies. Future studies should prioritize rigorous designs and the use of shared, standardized outcome measures to enhance comparability and evaluation.

## Figures and Tables

**Table 1:** Main characteristics of the included studies.

First Author Publication Date	Country	Year of Study	Study Design	Target of the Intervention	Type of Cancer	Target VPD	Multi-Component Intervention
Toleman M.S. 2015 [38]	UK	2012−2014	Quasi-Experimental, Pre-post evaluation	HCP	Any	Flu, Pneum.	Yes
Grivas P.D. 2016 [45]	USA	2011−2013	Natural Experimental, Pre-post evaluation	HCP + Patients	Any	Flu	Yes
Delacruz W. 2017 [44]	USA	2015−2016	Quasi-Experimental, Pre-post evaluation	HCP	Any	Pneum.	Yes
Church E.C. 2018 [43]	USA	2015	Quasi-Experimental, Pre-post evaluation	HCP + Patients	Chronic Lymphocytic Leukemia	Pneum.	Yes
Nipp R.D. 2018 [46]	USA	2017	RCT	Patients	Breast, gastrointestinal, or lung cancer.	Flu, Pneum.	No
Sitte J. 2018 [37]	France	2016−2017	Quasi-Experimental, Pre-post evaluation	Patients	Gastrointestinal	Flu, Pneum. (Diphtheria-Tetanus-Poliomyelitis, Hepatitis b, HPV)	No
Kelkar A.H. 2021 [41]	USA	2020−2021	Quasi-Experimental, Pre-post evaluation	HCP + Patients	Any	COVID-19	No
McGinnis J.M. 2021 [42]	Canada	2019−2020	Quasi-Experimental, Interrupted time series	HCP + Patients	Gynecologic	Flu, Pneum.	Yes
Tran V. 2021 [40]	France	2021	Quasi-Experimental, Pre-post evaluation	Patients	Any	COVID-19	No
Kiderlen T.R. 2022 [34]	Germany	2020−2021	Quasi-Experimental, Pre-post evaluation	HCP	Any	Flu, Pneum., Herpes Zoster and Hepatitis B	No
Narinx J. 2022 [39]	Belgium	2021	Quasi-Experimental, Post evaluation	Patients	Hematologic	COVID-19	Yes
Ganju P. 2023 [47]	India	2021−2022	Quasi-Experimental, Post evaluation	Patients	Multiple myeloma	COVID-19	No
Ozdemir N. 2023 [49]	Turkey	2019−2021	RCT	Patients	Any	Pneum.	Yes
Rivière P. 2023 [36]	France	2019−2020	Quasi-Experimental, Pre-post evaluation	HCP	Any	Flu, Pneum.	Yes
Shapiro Ben David S. 2023 [48]	Israel	2019−2021	Quasi-Experimental, Pre-post evaluation	HCP + Patients	Any	Pneum.	Yes

Abbreviations: HCP healthcare professional, Pneum. Pneumococcal disease, VPD vaccine preventable disease.

**Table 2:** Components of the included interventions.

First Author Publication Date	Patient Education	Patient Reminders	Patient Counselling	HCP Education	HCP Reminders	Other
Toleman M.S. 2015 [38]					X	Development of vaccination guidelines * Letter for primary care providers
Grivas P.D. 2016 [45]	X	X *			X *	
Delacruz W. 2017 [44]				X *		Recruitment of APRN to review vaccination status and prescribe vaccine if not done by oncologist
Church E.C. 2018 [43]		X *			X *	Pact between virtual clinic and primary care providers
Nipp R.D. 2018 [46]			X *			
Sitte J. 2018 [37]			X *			
Kelkar A.H. 2021 [41]	X *			X *		
McGinnis J.M. 2021 [42]	X *			X *		In-house vaccination program; pre-printed prescriptions for prescribing physicians
Tran V. 2021 [40]	X *					
Kiderlen T.R. 2022 [34]					X *	
Narinx J. 2022 [39]		X *	X			
Ganju P. 2023 [47]			X *			
Ozdemir N. 2023 [49]	X		X *			
Rivière P. 2023 [36]				X *		Development of a vaccination protocol
Shapiro Ben David S. 2023 [48]		X			X *	Preapproval for PCV13 is waived

Components marked with an asterisk (*) indicate the main component of the intervention. Abbreviations: APRN Advanced Practice Nurse Practitioner, HCP healthcare professional.

**Table 3:** Main outcomes and results of the included interventions.

First Author Publication Date	Primary Outcomes	Main Results
Toleman M.S. 2015 [38]	(1) Vaccination coverage: Flu (2) Vaccination coverage: Pneum.	(1) Flu: from 68.1% to 71.6% (*p* = 0.730) (2) Pneum. VC.: from 25% to 47% (*p* = 0.002)
Grivas P.D. 2016 [45]	Same day vaccination rate (Flu) (2 seasons)	Absolute increase in eligible adults vaccinated: 2.4% (*p* < 0.001) and 3.7% (*p* < 0.001)
Delacruz W. 2017 [44]	Vaccination coverage (Pneum.)	From 6.3% to 45.5% (*p* < 0.001)
Church E.C. 2018 [43]	Vaccination coverage (Pneum.)	Within 180 days, 62% received the vaccine. Significant difference in time to vaccination between pre and post-virtual clinic periods (log-rank test, *p* < 0.01).
Nipp R.D. 2018 [46]	(1) Vaccination coverage: Flu (2) Vaccination coverage: Pneum.	(1) Flu: intervention vs. control: 31.0% vs. 0.0% (*p* < 0.001) (2) Pneum.: intervention vs. control: 37.9% vs. 0.0% (*p* < 0.001)
Sitte J. 2018 [37]	Vaccination coverage (Pneum.)	From 10.1% to 87.5% (<0.001)
Kelkar A.H. 2021 [41]	(1) COVID-19 Vaccine intention (2) Changes in Beliefs and Perspectives on Vaccines against COVID-19	(1) From 71% to 82.5% (no *p*-value) (2) Increased agreeance with belief statements about vaccine effectiveness; vaccine safety; vaccine acceptance if recommended by a doctor; extra effort to receive a vaccine; and encouraging their family, friends, co-workers, and community to receive a vaccine. (no descriptive data) (*p* < 0.05)
McGinnis J.M. 2021 [42]	(1) Vaccination coverage: Flu (2) Vaccination coverage: Pneum.	(1) Flu: from 36% to a monthly mean of 67% (no *p*-value) (2) Pneum. VC.: from 5% to a monthly mean of 61% (no *p*-value)
Tran V. 2021 [40]	COVID-19 Vaccine intention	Among the cancer patients who did not intend to be vaccinated at baseline, 18.0% changed their mind (*p*-value)
Kiderlen T.R. 2022 [34]	Vaccination coverage (Pneum.)	Intervention vs. control: VC increase +21.5pp vs. −5.8pp (OR 4.94, 95% CI 1.76–13.83, *p* = 0.002)
Narinx J. 2022 [39]	Vaccination coverage (COVID-19)	Vaccination rates: 88.9% among patients of the intervention group; 76.3% in the general population (control) (Standardized Incidence ratio: 1.17; 95%CI 1.12−1.22, *p* < 0.001)
Ganju P. 2023 [47]	Vaccination coverage (COVID-19)	At least one dose: 86% (2 doses: 67%) (no comparisons, no *p*-value)
Ozdemir N. 2023 [49]	(1) Pneum. vaccination knowledge (higher score = higher number of correct answers) (2) Vaccination Attitudes Examination (VAX) score (higher score = higher negative attitude) (3) Vaccination coverage (Pneum.)	(1) Intervention vs. control: median 10 (IQR = 3) vs. 8 (IQR = 4) (*p* < 0.001) (2) Intervention vs. control: mean 33.09 (SD = 7) vs. 36.07 (SD = 6.5) (*p* = 0.007) (3) Intervention vs. control: 20.2% vs. 6.1% (*p* = 0.003)
Rivière P. 2023 [36]	(1) Vaccination coverage: Flu (2) Vaccination coverage: Pneum.	(1) Flu VC: from 42.6% to 55.1% (*p* = 0.08) (2) Pneum. VC: from 11.8% to 15.4%, (*p* =1)
Shapiro Ben David S. 2023 [48]	Vaccination coverage (Pneum.)	PCV13: from 11.9% to 52% (*p* < 0.001) PPSV23: from 39.4% to 57.1% (*p* < 0.001)

Abbreviations: CI Confidence Interval, IQR Interquartile Range, OR Odds Ratio, Pneum. Pneumococcal disease, SD Standard Deviation, VC Vaccination Coverage.

## Data Availability

No new data were created or analyzed in this study. Data sharing is not applicable to this article.

## Supplementary Materials

The following supporting information can be downloaded at https://www.mdpi.com/article/10.3390/vaccines13090964/s1, Supplementary File S1: Supplementary Methods. Search terms; Figure S1: Full flow diagram; Figure S2: Rob2 Evaluation; Table S1: Additional characteristics of the included studies; Table S2: Risk of bias of the quasi-experimental and natural experimental studies; Table S3: Quality of evidence evaluation. Ref. [67] is cited in Supplementary Materials.

## Abbreviations

The following abbreviations are used in this manuscript:

APRN	Advanced Practice Nurse Practitioner
ASCO	American Society of Clinical Oncology
EMR	Electronic Medical Record
GP	General Practitioner
HCP	Healthcare Professional
PICO	Population, Intervention, Comparison, Outcome
PRISMA	Preferred Reporting Items for Systematic reviews and Meta-Analyses
PROSPERO	International Prospective Register of Systematic Reviews
RCT	Randomized Controlled Trial
