# Supplementary material for: Strategies to Increase Vaccinations in Adult Cancer Patients: A Systematic Review
Source: Vaccines (Basel). 2025 Sep 11;13(9):964. doi: 10.3390/vaccines13090964 (PMC12474426; doi:10.3390/vaccines13090964)
Supplement: Supplementary file 1 [file vaccines-13-00964-s001.zip › vaccines-3821279-supplementary.pdf]

SUPPLEMENTARY FILE

Strategies to increase vaccinations in adult cancer patients: a systematic review

Authors

Giuseppina Lo Moro[1]*, Federica Golzio[1], Sara Claudia Calabrese[1], Giacomo Scaioli[1], Alessandro Basile[1], Roberta Siliquini[1,2°], Fabrizio Bert[1,3°]

Affiliations

[1]Department of Public Health and Pediatric Sciences, University of Turin, 10126 Turin, Italy
[2]Azienda Ospedaliera Universitaria City of Health and Science of Turin, 10126 Turin, Italy
[3]Collegium Medicum, University of Social Sciences, 90-113 Lodz, Poland

*Corresponding author: giuseppina.lomoro@unito.it
[°]Co-last authors: FB and RS contributed equally as last authors.

Table of contents

Supplementary File S1. Supplementary Methods. Search terms. 2
Figure S1. Full flow diagram. 4
Figure S2. Rob2 Evaluation. 5
Table S1. Additional characteristics of the included studies. 6
Table S2. Risk of bias of the quasi-experimental and natural experimental studies 8
Table S3. Quality of evidence evaluation 9

Supplementary File S1. Supplementary Methods. Search terms.

Query performed on November 24 2023:

PubMed:

(vaccin*[Title/Abstract] OR immuniz*[Title/Abstract] OR immunis*[Title/Abstract] OR “Vaccines”[Mesh] OR “Immunization”[Mesh] OR “Vaccination”[Mesh]) AND (cancer*[Title/Abstract] OR malignan*[Title/Abstract] OR carcin*[Title/Abstract] OR onco*[Title/Abstract] OR tumor*[Title/Abstract] OR tumour*[Title/Abstract] OR neoplasm*[Title/Abstract] OR “Neoplasms”[Mesh]) AND (hesit*[Title/Abstract] OR willing*[Title/Abstract] OR coverage*[Title/Abstract] OR uptake*[Title/Abstract] OR acceptanc*[Title/Abstract] OR adherence*[Title/Abstract] OR compliance*[Title/Abstract] OR refus*[Title/Abstract] OR Intent*[Title/Abstract] OR “Vaccination Refusal”[Mesh])

Scopus:

(TITLE-ABS(vaccin*) OR TITLE-ABS(immuniz*) OR TITLE-ABS(immunis*)) AND (TITLE-ABS(cancer*) OR TITLE-ABS(malignan*) OR TITLE-ABS(carcin*) OR TITLE-ABS(onco*) OR TITLE-ABS(tumour*) OR TITLE-ABS(tumor*) OR TITLE- ABS(neoplasm*)) AND (TITLE-ABS(hesit*) OR TITLE-ABS(willing*) OR TITLE-ABS(coverage*) OR TITLE-ABS(uptake*) OR TITLE-ABS (acceptanc*) OR TITLE-ABS(adherence*) OR TITLE-ABS(compliance*) OR TITLE-ABS(refus*) OR TITLE-ABS(intent*) OR TITLE-ABS({vaccination refusal}))

Embase:

(vaccin*:ab,ti OR immuniz*:ab,ti OR immunis*:ab,ti OR ‘vaccination’/exp OR ‘immunization’/exp) AND (cancer*:ab,ti OR malignan*:ab,ti OR carcin*:ab,ti OR onco*:ab,ti OR tumor*:ab,ti OR tumour*:ab,ti OR neoplasm*:ab,ti OR ‘neoplasm’/exp) AND (hesit*:ab,ti OR willing*:ab,ti OR coverage*:ab,ti OR uptake*:ab,ti OR acceptanc*:ab,ti OR adherence*:ab,ti OR compliance*:ab,ti OR refus*:ab,ti OR intent*:ab,ti OR ‘vaccination refusal’:ab,ti OR ‘vaccination refusal’/exp OR ‘vaccine hesitancy’/exp)

Query performed on February 20 2024:

PubMed:

((vaccin*[Title/Abstract] OR immuniz*[Title/Abstract] OR immunis*[Title/Abstract] OR “Vaccines”[Mesh] OR “Immunization”[Mesh] OR “Vaccination”[Mesh]) AND (cancer*[Title/Abstract] OR malignan*[Title/Abstract] OR carcin*[Title/Abstract] OR onco*[Title/Abstract] OR tumor*[Title/Abstract] OR tumour*[Title/Abstract] OR neoplasm*[Title/Abstract] OR “Neoplasms”[Mesh]) AND (“vaccination rate*”[Title/Abstract] OR “vaccine rate*”[Title/Abstract] OR “immunization rate*” [Title/Abstract] OR “immunisation rate*”[Title/Abstract])) NOT ((vaccin*[Title/Abstract] OR immuniz*[Title/Abstract] OR immunis*[Title/Abstract] OR “Vaccines”[Mesh] OR “Immunization”[Mesh] OR “Vaccination”[Mesh]) AND (cancer*[Title/Abstract] OR malignan*[Title/Abstract] OR carcin*[Title/Abstract] OR onco*[Title/Abstract] OR tumor*[Title/Abstract] OR tumour*[Title/Abstract] OR neoplasm*[Title/Abstract] OR “Neoplasms “[Mesh]) AND (hesit*[Title/Abstract] OR willing*[Title/Abstract] OR coverage*[Title/Abstract] OR uptake*[Title/Abstract] OR acceptanc*[Title/Abstract] OR adherence*[Title/Abstract] OR compliance*[Title/Abstract] OR refus*[Title/Abstract] OR Intent*[Title/Abstract] OR “Vaccination Refusal”[Mesh]))

Scopus:

((TITLE-ABS(vaccin*) OR TITLE-ABS(immuniz*) OR TITLE-ABS(immunis*)) AND (TITLE-ABS(cancer*) OR TITLE- ABS(malignan*) OR TITLE-ABS(carcin*) OR TITLE-ABS(onco*) OR TITLE-ABS(tumour*) OR TITLE-ABS(tumor*) OR TITLE-ABS(neoplasm*)) AND (TITLE-ABS(“vaccination rate*”) OR TITLE-ABS(“vaccine rate*”) OR TITLE- ABS(“immunization rate*”) OR TITLE-ABS(“immunisation rate*”))) AND NOT ((TITLE-ABS(vaccin*) OR TITLE- ABS(immuniz*) OR TITLE-ABS(immunis*)) AND (TITLE-ABS(cancer*) OR TITLE-ABS(malignan*) OR TITLE- ABS(carcin*) OR TITLE-ABS(onco*) OR TITLE-ABS(tumour*) OR TITLE-ABS(tumor*) OR TITLE- ABS(neoplasm*)) AND (TITLE-ABS(hesit*) OR TITLE-ABS(willing*) OR TITLE-ABS(coverage*) OR TITLE- ABS(uptake*) OR TITLE-ABS (acceptanc*) OR TITLE-ABS(adherence*) OR TITLE-ABS(compliance*) OR TITLE-ABS(refus*) OR TITLE-ABS(intent*) OR TITLE-ABS({vaccination refusal})))

Embase:

((vaccin*:ab,ti OR immuniz*:ab,ti OR immunis*:ab,ti OR ‘vaccination’/exp OR ‘immunization’/exp) AND (cancer*:ab,ti OR malignan*:ab,ti OR carcin*:ab,ti OR onco*:ab,ti OR tumor*:ab,ti OR tumour*:ab,ti OR neoplasm*:ab,ti OR ‘neoplasm’/exp) AND (“vaccination rate*”:ab,ti OR “vaccine rate*”:ab,ti OR “immunization rate*”:ab,ti OR “immunisation rate*”:ab,ti)) NOT ((vaccin*:ab,ti OR immuniz*:ab,ti OR immunis*:ab,ti OR ‘vaccination’/exp OR ‘immunization’/exp) AND (cancer*:ab,ti OR malignan*:ab,ti OR carcin*:ab,ti OR onco*:ab,ti OR tumor*:ab,ti OR tumour*:ab,ti OR neoplasm*:ab,ti OR ‘neoplasm’/exp) AND (hesit*:ab,ti OR willing*:ab,ti OR coverage*:ab,ti OR uptake*:ab,ti OR acceptanc*:ab,ti OR adherence*:ab,ti OR compliance*:ab,ti OR refus*:ab,ti OR intent*:ab,ti OR ‘vaccination refusal’:ab,ti OR ‘vaccination refusal’/exp OR ‘vaccine hesitancy’/exp))

Figure S1. Full flow diagram.

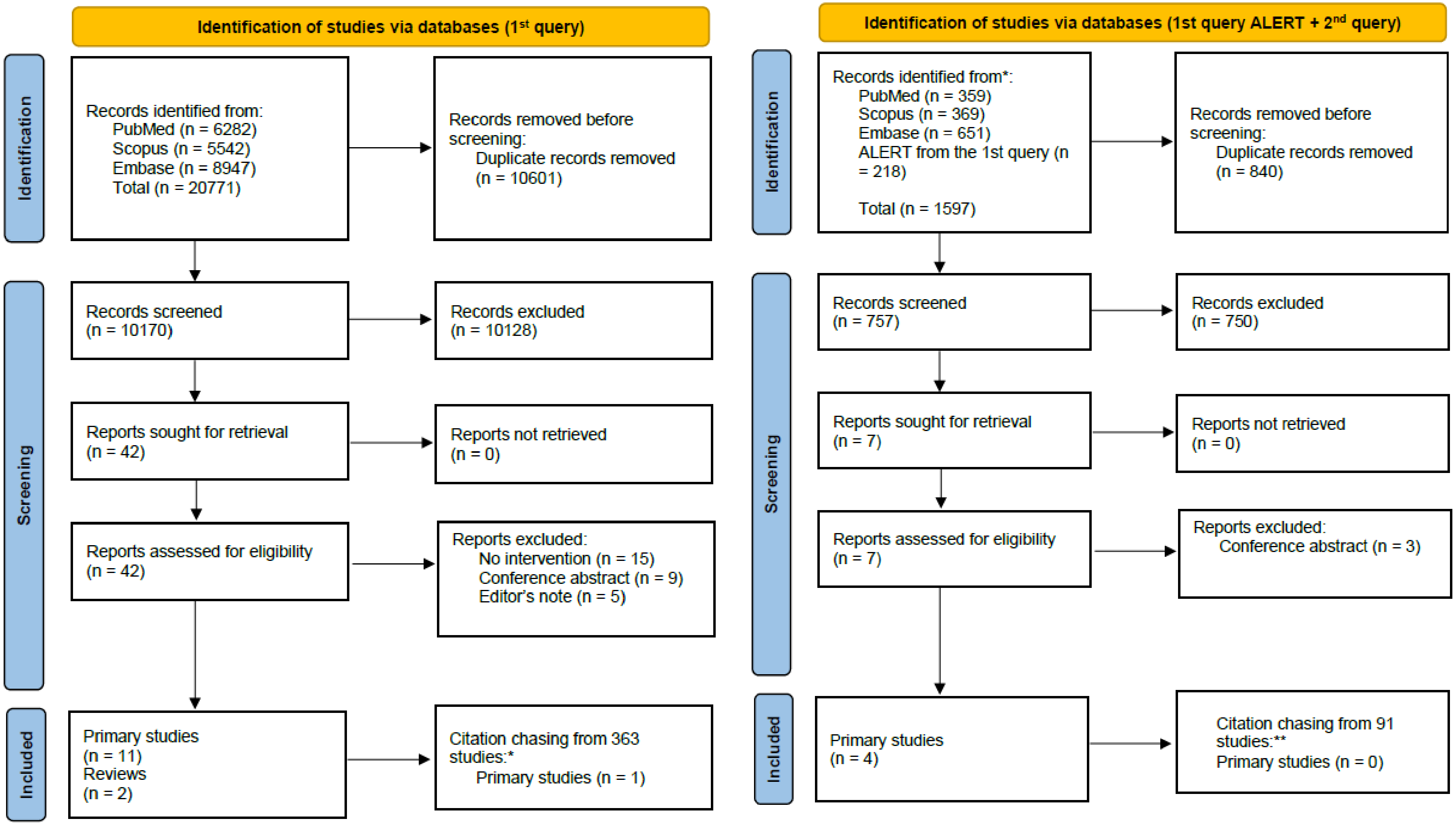

*From the 363 records identified through the reference lists of 11 primary studies and 2 reviews, 4 papers were selected after title and abstract screening. Full texts of these 4 papers were assessed: 1 duplicate, 2 excluded (no intervention), and 1 included. The reference list of the included paper (n=24) was also screened, but no additional studies were included.

**From the 91 records identified through the reference lists of 4 primary studies, 4 papers were selected after title and abstract screening. Full texts of these 4 papers were assessed: 3 duplicates, 1 excluded (no intervention). Thus, no additional studies were included.

Figure S2. Rob2 Evaluation.

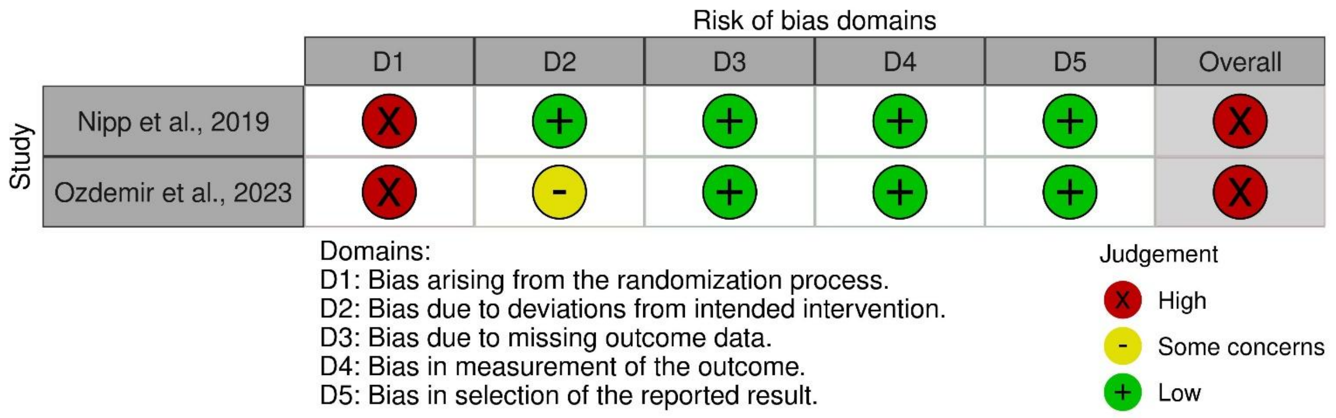

Created thanks to: McGuinness, LA, Higgins, JPT. Risk-of-bias VISualization (robvis): An R package and Shiny web app for visualizing risk-of-bias assessments. Res Syn Meth. 2020; 1- 7. https://doi.org/10.1002/jrsm.1411

Table S1. Additional characteristics of the included studies.

First Author & Publication date	Length of study	Target of the intervention	Number of participants target of the intervention	Information about HCPs	Information about patients	Type of cancer	Setting	Including paediatric patients	Control Group	Described as Quality Improvement intervention	Funding	Conflict of interests
**Toleman M.S. 2015**	28 months (from January 2012 to April 2014)	HCP	HCP: NA	Oncologists and specialist nurses	.	Any	Chemotherapy Day Unit in the Oxford Cancer Centre	No	NO	NO	NO	NO
**Grivas PD. 2016**	2 Influenza seasons (1 September 31 March), 14 months total	HCP + Patients	HCP: NA Patients: NA	Medical providers	Patients with cancer without documentation of influenza vaccination prior to their first outpatient appointment during the 2011–2012 and 2012–2013 influenza seasons	Any	Comprehensive Cancer Centre outpatient clinics	Yes	Historical and Contemporary controls (usual care)	YES	NO	N/A
**Delacruz W. 2017**	12 months (from April 2015 to March 2016)	HCP	HCP: 20 (10 physicians, 9 fellows, 1 advanced practice nurse practitioner)	Oncological staff	.	Any	A military/community medical oncology practice housed within a level 4 hospital.	No	NO	YES	N/A	NO
**Church EC. 2018**	6 months (started in August 2015)	HCP + Patients	HCP: NA Patients: 165	Nurses and providers	Patients with Chronic Lymphocytic Leukaemia who were not current for pneumococcal vaccines	Chronic Lymphocytic Leukaemia	Veteran Affairs medical centre	No	NO	NO	YES	NO
**Nipp R.D. 2018**	12 months (from January 2017 to October 2017 + 8 weeks)	Patients	Patients: Intervention: 29 Control: 31	.	Adults patients with cancer	Breast, gastrointestinal, or lung cancer.	Oncology clinic of an hospital	No	Usual care	NO	YES	NO
**Sitte J. 2018**	10 months (from December 2016 to September 2017)	Patients	Patients: 366 (27% with cancer)	.	Patients with gastrointestinal cancer or inflammatory bowel disease	Gastrointestinal cancer	Outpatient clinic of an hospital Gastroenterology department	No	NO	NO	NO	YES
**Kelkar AH. 2021**	17 days (from 31 December 2020 to 16 January 2021)	HCP + Patients	264 (participants), 105 (completed both surveys; 57% patients, 14% HCPs, 29% other)	Health care professionals	Patients with cancer (have cancer and actively receiving treatment, cancer survivor and not receiving treatment)	Any	Online	No	NO	NO	NO	NO
**McGinnis J.M. 2021**	12-month study period ending in March 2020. (patient telephone survey completed in May and June 2020)	HCP + Patients	HCP: 10 Patients: 219	Gynaecology staff	Patients who received a new line of chemotherapy for gynaecologic cancers over the study period	Gynaecologic cancers	Outpatient tertiary cancer centre.	No	NO	YES	YES	NO
**Tran V. 2021**	3 weeks	Patients	Patients: 3603 (263 with cancer)	.	Members of a e-cohort of patients with chronic	Any	Online	No	NO	NO	NO	NO

					conditions (including patients with cancer)							
**Kiderlen T.R. 2022**	12 months	HCP	HCP: NA	General Practitioners	.	Any	Outpatient clinics	No	Usual care	NO	YES	NO
**Narinx J. 2022**	8 months (from February 2021 to September 2021)	Patients	Patients: Intervention: 2379 Control: 883360 (Matched sample of the general population)	.	Immunosuppressed patients treated eligible for priority SARS-CoV2 vaccination (including patients with cancer)	Hematologic	Department of haematology at a tertiary academic hospital	No	Usual care	NO	NO	NO
**Ganju P. 2023**	14 months (from January 2021 to February 2022)	Patients	Patients: 178 (99% with multiple myeloma)	.	Patients with multiple myeloma and amyloidosis	Multiple myeloma	Urban tertiary care cancer hospital	No	NO	NO	NO	NO
**Ozdemir N. 2023**	20 months (from July 2019 to February 2021)	Patients	Patients: Intervention: 117 Control: 118	.	Patients with cancer	Any	Tertiary-care hospitals' medical oncology outpatient clinics	No	Usual care	NO	N/A	NO
**Rivière P. 2023**	4 months (from December to 2019 to March 2020)	HCP	NA	Medical staff	.	Any	Medical oncology departments of three hospitals	No	NO	NO	NO	NO
**Shapiro Ben David S. 2023**	2 years	HCP + Patients	HCP: NA Patients: NA	Staff: Physicians and nurses	Immunocompromised patients (including patients with cancer)	Any	Healthcare Service	No	NO	YES	YES	NO

Table S2. Risk of bias of the quasi-experimental and natural experimental studies

First Author & Publication date	PRIMARY OUTCOME	Is it clear in the study what is the "cause" and what is the "effect" (i.e. there is no confusion about which variable comes first)?	Was there a control group?	Were participants included in any comparisons similar?	Were the participants included in any comparisons receiving similar treatment/care, other than the exposure or intervention of interest?	Were there multiple measurements of the outcome, both pre and post the intervention/exposure?	Were the outcomes of participants included in any comparisons measured in the same way?	Were outcomes measured in a reliable way?	Was follow-up complete and if not, were differences between groups in terms of their follow-up adequately described and analysed?	Was appropriate statistical analysis used?
		**Item 1**	**Item 2**	**Item 3**	**Item 4**	**Item 5**	**Item 6**	**Item 7**	**Item 8**	**Item 9**
**Toleman M.S. 2015**	Vaccination coverage influenza	Y	N	Y	Y	N	Y	Y	N/A	Y
	Vaccination coverage pneumococcal vaccine					N	Y	Y	N/A	Y
**Grivas PD. 2016**	Same day vaccination rate (influenza)	Y	Y	N/A	N	N	Y	Y	Y	Y
**Delacruz W. 2017**	Vaccination coverage pneumococcal vaccine	Y	N	Y	N	N	Y	Y	Y	Y
**Church EC. 2018**	Vaccination coverage pneumococcal vaccine	Y	N	Y	Y	N	Y	Y	Y	Y
**Sitte J. 2018**	Vaccination coverage pneumococcal vaccine	Y	N	Y	Y	N	Y	Y	N	Y
**Kelkar AH. 2021**	COVID-19 Vaccination intention	Y	N	Y	Y	N	Y	Y	N	Y
	Change in belief					N	Y	Y	N	Y
**McGinnis J.M. 2021**	Vaccination coverage influenza	Y	N	Y	Y	Y	Y	Y	Y	Y
	Vaccination coverage pneumococcal vaccine					N	Y	Y	N	Y
**Tran V. 2021**	Vaccination intention	Y	N	Y	Y	N	Y	Y	Y	Y
**Kiderlen T.R. 2022**	Vaccination coverage pneumococcal vaccine	Y	Y	N/A	Y	N	Y	Y	Y	Y
**Narinx J. 2022**	COVID-19 Vaccination coverage	Y	Y	N/A	N/A	N	Y	Y	Y	Y
**Ganju P. 2023**	COVID-19 Vaccination coverage	Y	N	Y	Y	N	UNCLEAR	Y	Y	Y
**Rivière P. 2023**	Vaccination coverage influenza	Y	N	Y	Y	N	Y	Y	N	Y
	Vaccination coverage pneumococcal vaccine					N	Y	Y	N	Y
**Shapiro Ben David S. 2023**	Vaccination coverage pneumococcal vaccine	Y	N	Y	Y	N	Y	Y	N/A	Y

Table S3. Quality of evidence evaluation

PRIMARY OUTCOME: PNEUMOCOCCAL VACCINATION COVERAGE		
**Intervention**	**Results**	**Quality of Evidence Grades**
**Patient education and HCP education + In-house vaccination program; pre-printed prescriptions for prescribing physicians**		
Non-randomized studies		
McGinnis J.M. 2021	Pneum. VC.: from 5% to a monthly mean of 61% (no p-value)	Very low (mostly due to risk of bias)
**Patient education and patient counselling**		
RCT		
Ozdemir N. 2023	Intervention vs control: 20.2% vs 6.1% (p=0.003)	Very low (mostly due to risk of bias and imprecision)
**Patient and HCP reminders**		
Non-randomized studies		
Church EC. 2018	Within 180 days, 62% received the vaccine. Significant difference in time to vaccination between pre and post-virtual clinic periods (log-rank test, p < 0.01).	Very low (mostly due to risk of bias)
Shapiro Ben David S. 2023	PCV13: from 11.9% to 52% (p<0.001); PPSV23: from 39.4% to 57.1% (p<0.001)	
**Patient counselling**		
RCT		
Nipp R.D. 2018	Pneum.: intervention vs control: 37.9% vs. 0.0% (p<0.001)	Very low (mostly due to risk of bias and imprecision)
Non-randomized studies		
Sitte J. 2018	From 10.1% to 87.5% (<0.001)	
**HCP education + dedicated professionals**		
Non-randomized studies		
Delacruz W. 2017	From 6.3% to 45.5% (p<0.001)	Very low (mostly due to risk of bias)
**HCP education + development of a vaccination protocol**		
Non-randomized studies		
Rivière P. 2023	Pneum. VC: from 11.8% to 15.4%, (p=1)	Very low (mostly due to risk of bias)
**HCP reminders + Development of vaccination guidelines, Letter for primary care providers**		
Non-randomized studies		
Toleman M.S. 2015	Pneum. VC.: from 25% to 47% (p=0.002)	Very low (mostly due to risk of bias)
**HCP reminders**		
Non-randomized studies		
Kiderlen T.R. 2022	OR 4.94, 95% CI 1.76–13.83, p=0.002	Very low (mostly due to risk of bias and imprecision)

PRIMARY OUTCOME: FLU VACCINATION COVERAGE		
**Intervention**	**Results**	**Quality of Evidence Grades**
**Patient education and HCP education**		
Non-randomized studies		
McGinnis J.M. 2021	Flu: from 36% to a monthly mean of 67% (no p-value)	Very low (mostly due to risk of bias)
**Patient counselling**		
RCT		
Nipp R.D. 2018	Flu: intervention vs control: 31.0% vs. 0.0% (p<0.001)	Very low (mostly due to risk of bias and imprecision)
**HCP education + development of a vaccination protocol**		

Non-randomized studies		
Rivière P. 2023	Flu VC: from 42.6% to 55.1% (p=0.08)	Very low (mostly due to risk of bias)
**HCP reminders + Development of vaccination guidelines, Letter for primary care providers**		
Non-randomized studies		
Toleman M.S. 2015	Flu: from 68.1% to 71.6% (p=0.730)	Very low (mostly due to risk of bias)

PRIMARY OUTCOME: COVID-19 vaccination coverage

**Intervention**	**Results**	**Quality of Evidence Grades**
**Patient reminder and counselling**		
Non-randomized studies		
Narinx J. 2022	Vaccination rates: 88.9% among patients of the intervention group; 76.3% in the general population (control) (Standardized Incidence ratio: 1.17; 95%CI 1.12-1.22, p<0.001)	Very low (mostly due to risk of bias)
**Patient counselling**		
Non-randomized studies		
Ganju P. 2023	At least one dose: 86% (2 doses: 67%) (no comparisons, no p-value)	Very low (mostly due to risk of bias)
